# Age-related changes in motor planning for prior intentions: a mouse tracking reach-to-click task

**DOI:** 10.3389/fpsyg.2024.1323798

**Published:** 2024-03-18

**Authors:** Shujing Zhang, Kate Wilmut, Kaiyu Zhang, Shan Wang

**Affiliations:** ^1^Global Health Research Center, Duke Kunshan University, Kunshan, China; ^2^Division of Natural and Applied Sciences, Duke Kunshan University, Kunshan, China; ^3^Department of Psychology, Health and Professional Development, Oxford Brookes University, Oxford, United Kingdom; ^4^Department of Geriatrics, The First People's Hospital of Kunshan, Kunshan, China; ^5^Department of Psychology, University of Bath, Bath, United Kingdom

**Keywords:** motor planning, prior intention, aging, computer mouse cursor tracking, kinematic analysis

## Abstract

When we complete sequential movements with different intentions, we plan our movements and adjust ahead. Such a phenomenon is called anticipatory planning for prior intentions and is known to decline with age. In daily life activities, we often need to consider and plan for multiple demands in one movement sequence. However, previous studies only considered one dimension of prior intentions, either different types of onward actions or different precisions of fit or placement. Therefore, in this study, we investigated anticipatory planning for both extrinsic (movement direction) and intrinsic (fit precision) target-related properties in a computer-based movement task and analyzed the computer cursor movement kinematics of both young and older adults. We found that older people consider and adjust for different properties step-by-step, with movement direction being considered as a prior intention during reach movement and fit precision as a motor constraint during drop movement. The age-related changes in the completion of onward actions are constrained by one’s general cognitive ability, sensorimotor performance and effective motor planning for prior intentions. Age-related decline in motor planning can manifest as counterproductive movement profiles, resulting in suboptimal performance of intended actions.

## 1 Introduction

Anticipatory motor planning refers to one’s motor preparation and movement selection that plays a vital role in shaping our ability to perform daily activities, ranging from pouring a cup of tea (Rosenbaum et al., 2001) to crossing a busy street (Geraghty et al., 2016). As individuals advance in age, the once seemingly effortless orchestration of movement planning and execution may become more demanding. For example, age-related alteration in hand movements often manifests as reduced precision and/or elongated movement time, which has been found to be associated with less effective anticipatory planning (Stöckel et al., 2017; Wilmut and Wang, 2019; Wang et al., 2020). One way to study anticipatory planning is to observe alterations in one’s initial action as a function of the subsequent action that is needed – getting ready for what is anticipated. For example, when we need to put a bottle on the top shelf of a cupboard, we tend to grasp a lower part of the bottle compared to when we need to leave the bottle on the floor. In this case, if the grasp height was altered depending on the subsequent placement height, such alterations are considered evidence of anticipatory planning.

One phenomenon that has been focused on in human movement research is planning for prior intentions (Jeannerod, 2006). Planning for prior intentions is often studied in a two-step movement task, in which the second movement varies with different demands, and the first movement remains unchanged. Thus, alterations in the first step of the movement as a function of the second movement demands are indicative of planning for the “prior intention” (Egmose and Køppe, 2018). For example, in a pioneer study conducted by Marteniuk et al. (1987), participants were asked to reach to grasp a disk and fit it into a hole or reach to grasp the same disk and throw it into a container, where the initial “reach-to-grasp” was identical across conditions, and the only difference was the prior intention of “fit” vs. “throw.” Marteniuk et al. (1987) observed a longer deceleration period in the initial reach movement preceding a subsequent “fit” than a “throw,” which is evidence of planning for prior intention requiring a higher precision (fit) than a lower precision (throw). Indeed, such adjustment in the reach movement leads to better performance in completing the onward actions. Children, whose motor planning ability do not mature until age 11–12, show less early motor adjustment for prior intentions and result in less smooth completion of the onward actions (Wilmut et al., 2013).

It is known that planning for prior intentions also declines with advanced age (Bennett and Castiello, 1994; Weir et al., 1998; Wilmut and Wang, 2019). A lack of adjustment in the reach movement (i.e., similar deceleration period) for different prior intentions among older adults is associated with a longer adjustment time for completing the onward actions. Previous research observed the best motor planning performance among young adults in their 20s and 30s, who can differentiate different types of onward actions and different levels of difficulty of the same type of action (Wilmut and Wang, 2019). For example, young people (20s and 30s) used more time to decelerate their reach movement for lift than tight place than loose place than throw, as onward actions, and led to smoother completion of the final movement. The earliest sign of prior intention planning decline became observable from mid-age (40s and 50s), where individuals only adjusted for different types of actions (lift vs. place vs. throw) but not different levels of difficulty (tight vs. loose place). Such planning ability further dropped with advanced aging in the 60s and 70s, where the initial reach movement was not adjusted for different onward actions at all (Wilmut and Wang, 2019). From the perspective of assessment, planning for prior intention seems to be a paradigm that is sensitive for early aging detection and advanced age-related decline.

Studies so far commonly considered prior intention by manipulating the level of difficulty of the onward actions in one dimension, from simpler to more challenging (Egmose and Køppe, 2018). For example, studies considered different types of movement that introduce different levels of demand, such as lift versus fit or place versus throw, where “lift” is often the most demanding and “throw” the least. Among fit or place movements, levels of precision were considered, depending on the size ratio between the object being manipulated and the target container, where a tight fit is assumed to be more demanding and requires more adjustment and better control than a loose fit. In Wilmut and Wang (2019), different types of onward actions (e.g., lift vs. throw) and different levels of precision requirement (e.g., tight vs. loose fit) were considered in one dimension in terms of how challenging the movement is. However, in everyday actions, the levels of movement difficulty are not isolated to one factor or one dimension but across multiple factors or dimensions and sometimes need to be considered simultaneously. For example, we may need to fit objects to containers of different sizes at a higher or a lower-level shelf, where both the fit precision and movement height need to be considered. In this case, do we differentiate multiple aspects of movement demands of prior intentions (e.g., precision and height) and adjust our movements differently, for example, planning for one factor after another? Or do multiple factors have an additive effect on the movement difficulty, and the reach movement adjustment is a function of the additive/total onward action difficulty, regardless of which aspect (e.g., height or precision) is being considered? Moreover, will such planning alter as we grow older? For example, will young people with better motor capacity plan everything in one go, and older people plan different demands step by step as a strategy to compensate for the cognitive slow down or less optimal motor control and reduced movement precision?

To answer these questions, this study presents a computer-based movement task to investigate the anticipatory planning for multiple prior intentions in one movement sequence and the age-related changes. The task paradigm is adopted from the real-world reach-to-grasp task by Marteniuk et al. (1987). Our computerized reach-to-click task also requires a two-step movement, in which participants need to “reach-to-click” on an object and then drop it into a target circle using a computer mouse. The prior intentions were manipulated using target circles of different sizes (i.e., tight fit, medium fit, and loose fit) and target locations on different sides (i.e., left vs. right-forward movement). Few studies considered the effect of movement direction as a prior intention. Studies of computer mouse ergonomics examined the effect of movement direction in single-step mouse tapping or pointing tasks and found that although moving horizontally left is easier than moving horizontally right, moving left forward/upward is more difficult than moving right forward/upward, showing longer movement durations and lower accuracy (Dillen et al., 2005; Hertzum and Hornbæk, 2010; Stewart et al., 2013). Similar to Wilmut and Wang (2019), in the current computerized task, the direction of movement will be diagonal rather than horizontal. Thus, the left and right directions here refer to left forward/upward (northwest) and right forward/upward (northeast) movements. Thus, in this study, we considered both target size and target side as prior intentions, where in terms of target size difficulty, tight is more difficult than medium than loose, and in terms of target side, left-forward is more difficult than right-forward.

Studies of movement kinematics so far have relied on motion-tracking systems. A possible low-cost alternative is tracking computer users’ mouse cursor trajectories, which is a new window into human (hand) movement. In a computer-based experimental set-up, participants are able to interact with the programmed experiment using their mouse, which resembles what they would do using their hands and arms in a real-world environment, e.g., a lab setting. Their hand or mouse movement could be accessed and recorded in a means of the cursor trajectories, which are dynamic, updated continuously, and presumably reflecting how the underlying mental processing unfolds. While few studies used computer mouse tracking to study movement planning, it has been used in a wide range of domains in the psychological and behavioral sciences (Freeman, 2018; Schoemann et al., 2021). More recently, the computer mouse has been used as a valid tool to study motor adaptation and sensorimotor learning (Lee et al., 2016; Avraham et al., 2021; Listman et al., 2021; Tsay et al., 2021, 2023; Balestrucci et al., 2022; Donovan et al., 2022; Weightman et al., 2022) across a wide age range from 18 to 70 (Tsay et al., 2023). It has been found such data collected through computer mouse are reliable, valid, and able to reproduce classic findings as in the literature examined 3D real-world hand movements, even among children between 9 and 16 (Malone et al., 2023), older people with and without Parkinson’s disease (Tsay et al., 2022) and individuals with impaired visual function (Tsay et al., 2023).

Thus, we expected the data collected through a computer mouse for the “reach-to-click” task to reproduce the effect of anticipatory planning for prior intentions. Despite the fact that initial demands of the reach component were identical across conditions, we expected to see adjustments in the reach movements according to the demands of the onward action. According to the precision hypothesis (Marteniuk et al., 1987), increased action demands would lead to an elongated deceleration phase. Such an effect can arise as a prior intention, where highly demanding final action leads to elongated initial movement deceleration. Thus, we expected to see an elongated deceleration of reach movement preceding an onward action of a higher-level difficulty. The interactive influences of these two factors are still to be seen. In terms of age-related changes, studies of real-world hand movement found that, with advanced age, older people showed less adjustment during reach movement for different prior intentions than young people, which accordingly led to less efficient onward actions (Weir et al., 1998; Wilmut and Wang, 2019). In this study, we hypothesized that young adults, compared to older adults, would show greater differentiation for onward intentions during the reach phase. Such differentiation may manifest as a longer deceleration period for more challenging onward intentions (e.g., a tight fit). Furthermore, we anticipated that young adults would perform the drop actions more smoothly, characterized by fewer discontinuities in the velocity profile, resulting in a shorter period of final adjustments compared to older adults.

To further consider possible constraints of age-related changes in the planning for prior intentions, we also examined individuals’ general cognitive function and sensorimotor function (eye-hand coordination), which are both considered key to movement control and planning (Stöckel et al., 2017; Wang et al., 2020) and both sensitive to aging (Guan and Wade, 2000; Deary and Der, 2005). Recent research found that the capacity for anticipatory motor planning is shaped by cognitive abilities, which appear to play a crucial role in compensating for the decline associated with aging. For example, Stöckel et al. (2017) found that age-related differences in motor planning performance can be accounted for by cognitive functions, including processing speed, response planning, and cognitive flexibility. Wang et al. (2020) found that, in addition to individuals’ physical constraints, age-related decline in anticipatory planning can be accounted for by one’s executive function inhibition, working memory span and motor imagery. Given the wide range of cognitive abilities and possible overlaps among them, in this study, we considered a simple measure of cognitive and sensorimotor function, namely choice reaction time and eye-hand coordination. According to the processing speed hypothesis of cognitive aging, reaction time elongates with age, mainly due to the slowing down of information processing, and accounts for a substantial proportion of the age-related variance (or decline) in higher cognitive functions such as memory, reasoning, and executive functions. Choice reaction time is, therefore, considered the best parsimonious measure that accounts for the core ability in heterogeneous cognitive tasks (Deary and Der, 2005). It is also evident that eye-hand coordination is one of the most important sensorimotor functions that declines with advancing age (Guan and Wade, 2000) and plays a pivotal role in goal-directed movements (Binsted et al., 2001). We thus expected to see an age-related decline in eye-hand coordination and choice reaction time, and one’s reach-to-click task performance would be constrained by their general cognitive and sensorimotor functions.

## 2 Methods

### 2.1 Design

A 3 × 2 × 2 mixed design for repeated measures was used in the study, with the within-subject variables being Target Side (left vs. right) and Target Fit (tight vs. medium vs. loose), and the between-subject variable being Age Group (young vs. older).

### 2.2 Participants

A prior power analysis with a medium effect size (*f* = 0.25) and power of 0.95 for the repeated measures design showed that an estimated sample size of 28 is needed for each group. A total of 56 young participants (43 females) ranging from 18 to 22 years of age were recruited from Duke Kunshan University with an average of 19.09 years (*sd* = 1.05). Thirty-six older participants (24 females) ranging from 40 to 68 years of age were recruited from Duke Kunshan University and Kunshan local community with an average of 55.86 years (*sd* = 7.26). All the older participants completed the Chinese version of the Mini-Mental State Examination (MMSE; Li et al., 2016). According to their educational level, all the older participants have normal cognitive abilities (Katzman et al., 1988). For older participants with one 6 years of education (*n* = 10; age ranged from 45 to 68 years with a mean of 56.1 years), their MMSE total scores ranged from 19 to 29 with a mean of 25.3 (optimal threshold 19/20; Li et al., 2016). For those with 7 years or more of education (*n* = 24; age ranged from 40 to 68 years with a mean of 55.54 years), their MMSE total scores ranged from 26 to 30 with a mean of 28.5 (optimal threshold 23/24; Li et al., 2016). All the participants reported to have normal or correct-to-normal vision and are free from any known neurological or psychological disorders. Participants’ handedness was measured using the Edinburgh Handedness Inventory – Short Form (Veale, 2014). Eight young participants are ambidextrous. The rest of the 48 young participants and all the older participants are right-handed. In addition, all the participants reported being right-handed with computer mouse use. The study was reviewed and approved by the Duke Kunshan University Institutional Review Board (Protocol #2020SW0048).

### 2.3 Tasks and measures

#### 2.3.1 Movement planning task

**Figure 1:**
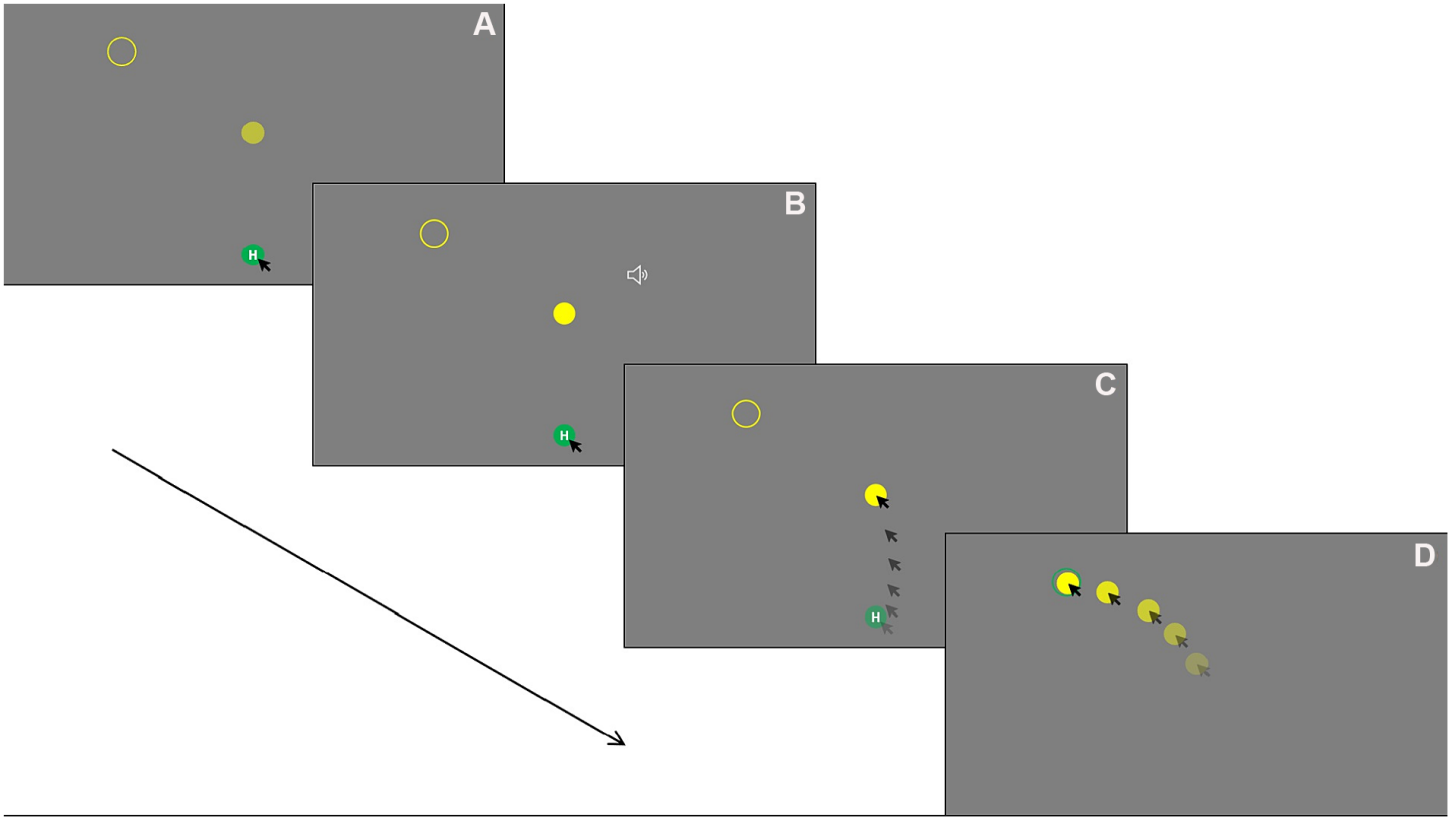
Mouse tracking task procedure using left side tight fit as an example: **(A)** Holding down the mouse key on the home button for a random interval between 500 and 1,500 ms. **(B)**. Once the holding time elapsed, the central disk blinks once with a brief audio tone played for 300 ms simultaneously to notify the participant to start. **(C)** The participant moving the cursor from the home button toward the central disk and click on it (reach phase). **(D)** Dragging the central disk toward the target circle and drop it (drop phase).

In the current study, a modified, computer-based reach-to-click task was designed and programmed using PsychoPy 3 and executed on a Dell Latitude 7,490 laptop with Windows 10. Participants sat at a comfortable distance of approx. 60 cm from a 23-inch LCD screen. The monitor was operated at a resolution of 1920 × 1,080 pixels with a refresh rate of 60 Hz. The display consisted of a green home button at the bottom center of the screen [coordinates = (0, −400)], a yellow disk at the center [coordinates = (0, 20)], and a yellow target circle at the upper-left corner [coordinates = (−280, 300)] or the upper-right corner [coordinates = (280, 300)] of the screen (see Figure 1). Both the home button and the disk are 30 pixels in diameter. There were three sizes of the target circle: 34 pixels (tight-fit), 60 pixels (mid-fit) and 90 pixels (loose-fit) in diameter, with one shown at a time. The three target circle sizes were determined according to the disk size, which is constant of 30 pixels, and increased by integer factors. This is in line with previous studies of this type (Johnson-Frey et al., 2004; Gigliotti et al., 2020). The tight-fit target circle was slightly larger than the disk (34 pixels) to allow fitting, which is similar to Marteniuk et al. (1987) and Weir et al. (1998). The ratio of the distance between objects in the current display is the same as the setup used in a previous study by the authors (Wilmut and Wang, 2019).

Participants were asked to fixate on the screen while holding down the mouse key on the home button with the index finger of the dominant hand. Their non-dominant hand rested comfortably on the table. After a random interval from 500 to 1,500 ms, which was designed to reduce anticipatory error, the disk blinked once with a brief audio tone played for 300 ms simultaneously to notify the participant to move, click on the disk, and then drag and drop it into the target circles. Once the disk was fit into the target, the target circle changed color to green to inform the participant of a successful fit. To complete a trial, the participant needed to release the mouse button to drop the disk into the target circle. If a participant released home button too early before holding the time elapsed, the trial would re-start. If a participant’s reach movement time was greater than 2,000 ms (from the onset cue of disk blink and audio tone to clicking on the central target), the trial re-started. Participants were instructed to move the mouse cursor within the screen area and avoid overshooting. A warning tone with text information (‘Please keep mouse cursor in the screen area’) would be provided if the mouse cursor reached the edge of the screen during a trial, and the trial would re-start. In all these cases, the initial unsuccessful trial would not be recorded. A sample trial is illustrated in Figure 1. In this paradigm, the movements were considered in two phases: the initial reach-to-click movement is from the onset of the movement from the home button to clicking on the central disk, which is referred to as “reach” in this study, and the onward transport-to-drop movement is from moving the central disk from its initial location to successfully drop it into the target circle, which is referred to as “drop” in this study.

The process was explained to the participant before the start of the experiment, and a step-by-step demo was provided. Each participant completed two blocks of the task, with one block showing target circles at the upper left and the other on the upper right. There were 30 trials in each block, with each size of the target circle appearing 10 times. The order of the blocks was counterbalanced across participants. Each participant had to successfully complete three practice trials before proceeding to the main task of each block. Responses were made using a wired laser computer mouse (Dell MS116, movement resolution: 1,000 dpi). The enhanced computer mouse cursor acceleration was disabled during the task, resulting in a hand-to-cursor ratio of approx. 0.3 cm per 100 pixel. The mouse cursor movement trajectories were recorded at a sampling rate of 60 Hz, identical to the monitor refreshing rate. The experiment took place in a quiet lab with normal lighting. A stable gray background was used throughout the task.

#### 2.3.2 Daily computer mouse use

We asked participants to rate their daily mouse use from 1 to 5, with 1 = “I rarely use a mouse,” 2 = “Less than 1 h per day,” 3 = “1–2 h per day,” 4 = “2–4 h per day,” 5 = “More than 4 h per day.” Indeed, there is a significant difference between young and older participants’ daily mouse use [*t*(81.77) = −2.41, *p* = 0.018], where young participants (mean rating = 2.16, *sd* = 1.57) used the mouse more often than older participants (mean rating = 1.45, *sd* = 1.17).

#### 2.3.3 Sensorimotor function – pursuit rotor task

One’s eye-hand coordination was assessed using the computerized pursuit rotor task (Willingham et al., 1995) within the Psychology Experiment Building Language (PEBL) battery (Mueller and Piper, 2014). During the task, participants were asked to use the computer mouse to track a small red disk moving steadily around a circular path and to try to keep the cursor on the path at all times. The same setting was used for all the participants, with the path radius being 253 pixels and the 25 pixels radius red disk moving along the path at a speed of 0.13 rotations/s. All the participants completed four trials using their preferred hand of mouse use (i.e., right hand), with each trial taking 15 s to complete. The proportion of time on target (out of 15 s) was computed and used as a measure of individuals’ sensorimotor function. An independent t-test revealed a significant difference [*t*(86) = 15.76, *p* < 0.001, Cohen’s *d* = 3.45], where young participants (mean = 0.78, *sd* = 0.10) had a larger proportion of time on the target than older participants (mean = 0.27, *sd* = 0.20).

#### 2.3.4 Cognitive function – choice reaction time

Participants’ reaction time was measured using the “Go” trials in the training phase of a Stop Signal Task. As a part of a larger project, participants completed the Psytoolkit Stop Signal Task (Stoet, 2010). In the training phase of the task, participants were presented with a green arrow pointing to either the left or the right for 500 ms as a “go” signal. Their task was to respond to the signal by pressing either the “left” or the “right” arrow key on the keyboard within the 500 ms time window. Participants either successfully completed 20 consecutive trials or have done all 50 trials. Choice reaction time (CRT) was calculated from the onset of the “Go” signal to the moment a key-pressing was made. SRTs of correct responses were recorded, and RTs that fell outside the range of the mean ± 2.5 *sd* were excluded from the analysis. An independent *t*-test revealed a significant age difference [*t*(85) = −8.75, *p* < 0.001, Cohen’s *d* = −1.93], where young people’s RT (mean = 365 ms, *sd* = 25) was shorter than older participants (mean = 414 ms, *sd* = 26).

### 2.4 Data analysis

#### 2.4.1 Kinematic analysis

**Figure 2:**
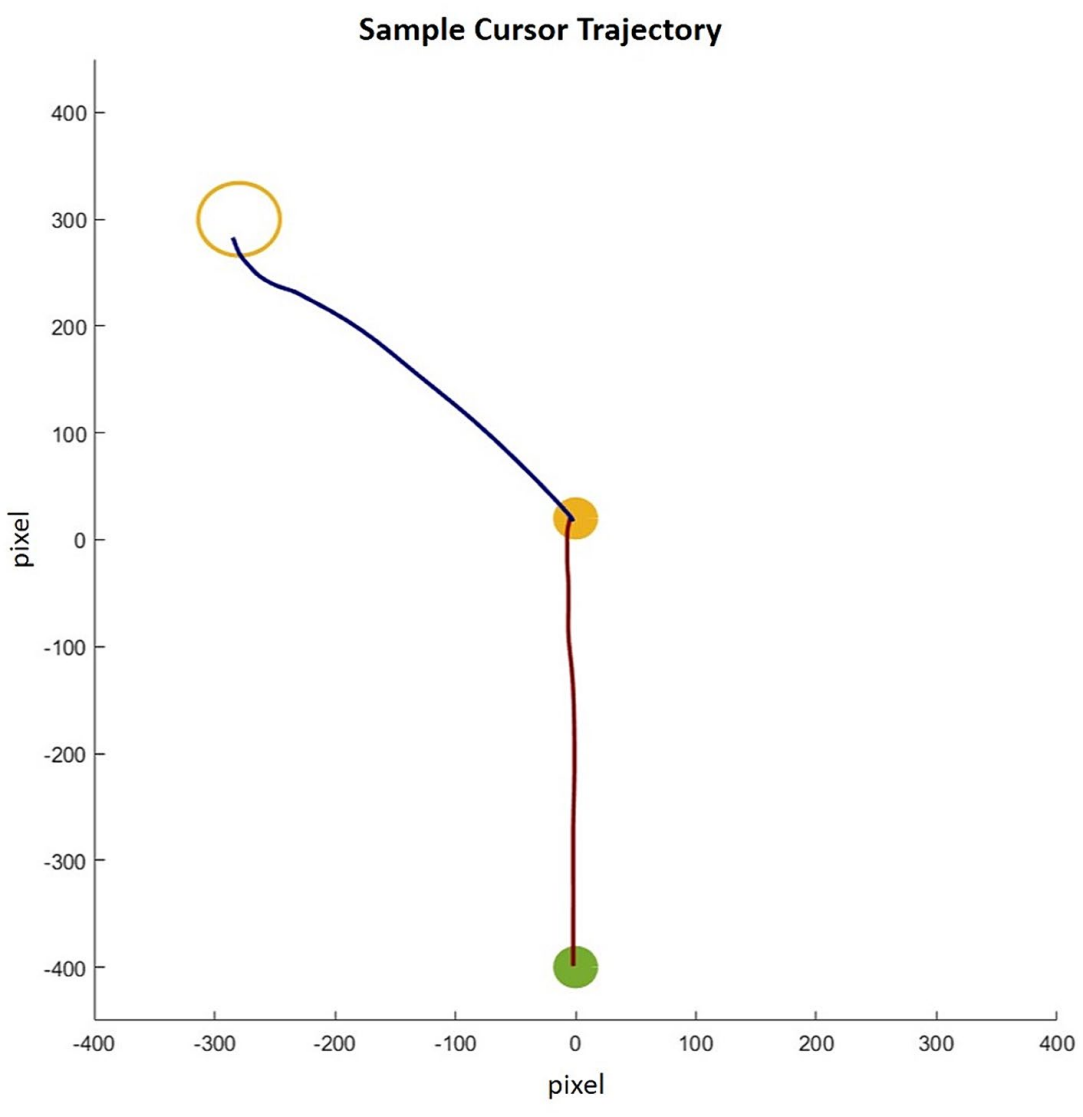
Sample cursor trajectory for a left-side medium-size drop. The red trajectory line shows reach movement and the blue trajectory line shows the drop movement.

Data from 54 young and 34 older participants were included in the analysis. Four participants (two young and two older) were excluded from the analysis due to missing data and recording errors. The computer mouse movement data were filtered with an optimized Woltring filter with a low-pass cutoff frequency of 10 Hz and analyzed using tailored MATLAB routines. A sample cursor trajectory is illustrated in Figure 2. The displacement data were differentiated to gain the instantaneous tangential velocity at each time point of the movement, and the acceleration was gained by differentiating the velocity data. Similar to Wilmut and Wang (2019), the movement onset and offset were defined as the time point at which the mouse cursor velocity surpassed and fell below 3% of the peak velocity of the movement, respectively. These time points of each trial were first detected automatically and then inspected visually.

**Figure 3:**
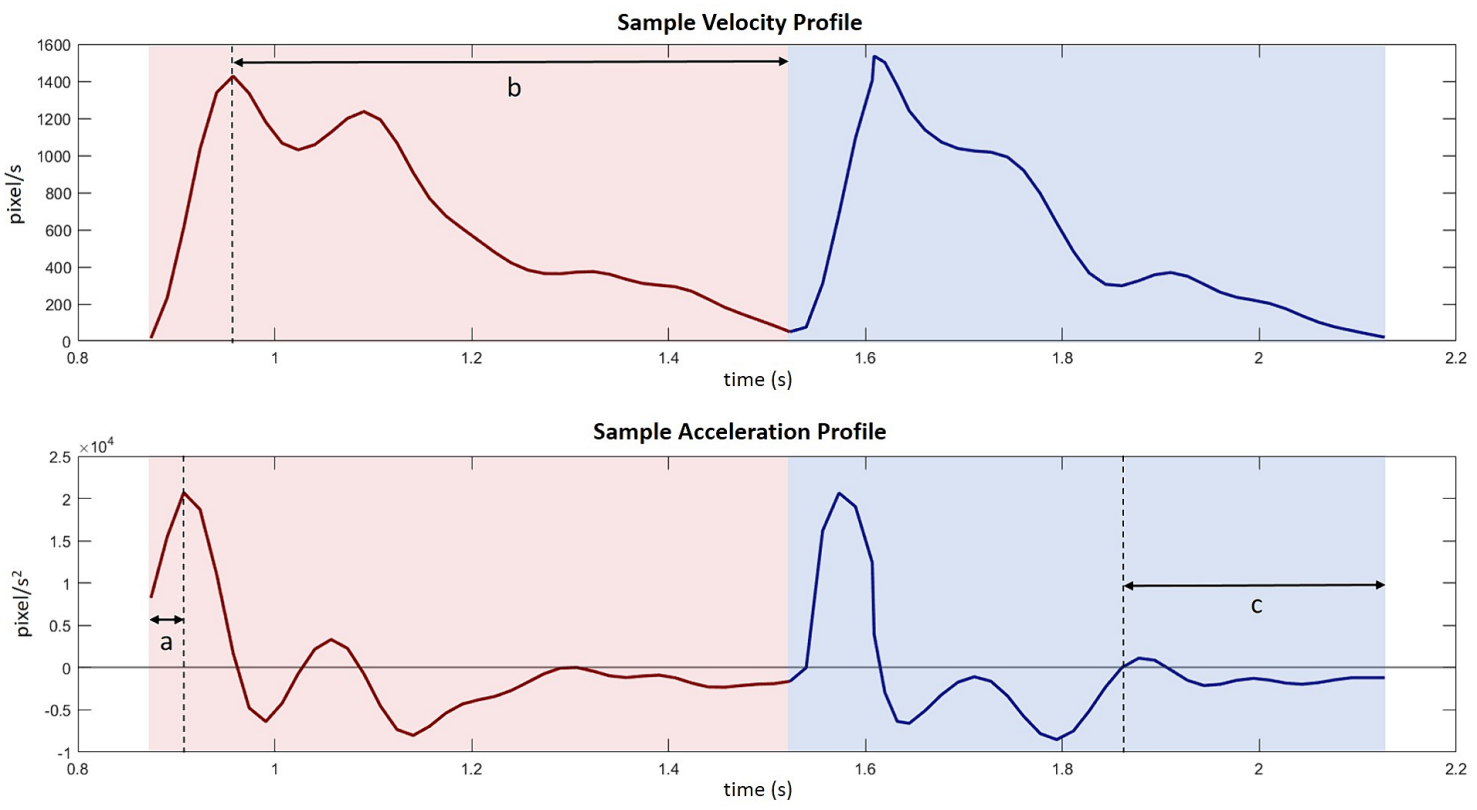
Sample velocity and acceleration profile with several key kinematic measures: (a) time to peak acceleration during reach movement, (b) deceleration time during reach movement, (c) adjustment time before finishing the drop movement.

Data were first screened for movement success and recording errors. Across all the participants, a total of 427 trials (194 young 6.0% and 233 older 11.4%) were identified as having unsuccessful movement trajectories or recording errors and excluded from further analysis. While the final drop was successful, the unsuccessful trials often had zig-zagged trajectories with multiple forward and backward, leftward and rightward, S-shaped, or repeated swiping-through movements. These back-and-forth movement trajectories can be due to the ‘loss’ of target (caused by unintentional mouse button releasing during the drop movement) or other unknown reasons. Thus, the kinematic measures of these trials did not necessarily reflect the features of the intended movement and were not included in the analysis. These unsuccessful trials were first identified automatically by detecting whether the trajectory moved in the opposite direction from the aiming target for more than a third of the distance along the connecting line between the start and the end of the reach or drop movement. For instance, during the reach phase, the ideal trajectory was to move upright, if the cursor trajectory moved backward along the *y*-axis for more than a third of the length of the distance between the home button and the central disk, then the trial was likely to have multiple back-and-forth movements and identified as unsuccessful. After automated detection, we visually inspected all the trial trajectories and plotted key kinematic measures to screen trials with extremely long movement durations or path lengths. The data were also screened for response latency (from the onset of the audio cue to the initiation of the reach movement). A total of 109 trials (84 young 2.8% and 25 older 1.4%) were excluded due to RTs less than 100 ms or longer than the mean + 2.5 *sd*. Average response latency toward audio stimulus ranges from 140 to 160 ms (Welford, 1980), so in the current study, 100 ms was used as a lower cut-off. A series of kinematic measures were calculated to depict the temporal and spatial features of the movement at the initial reach phase (from the home button to the disk) and the following drop phase (from the disk to the target circle). See Figure 3 for a sample velocity and acceleration profile. In both phases, we calculated: (a) movement duration (ms), the time between movement onset and movement offset; (b) peak velocity (px/s), maximum velocity between movement onset and offset; (c) peak acceleration (px/s^2^), maximum acceleration between movement onset and offset; (d) time to peak acceleration (%), the time from movement onset until the point of maximum acceleration prior to peak velocity as a percentage of movement duration (Figure 3 – a); (e) deceleration time (%), the time between peak velocity and movement offset as a percentage of movement duration (Figure 3 – b); (f) path length, the accumulated traveled distance from the start to the end of the movement. For reach movement, (g) the maximum path deviation on the *x*-coordinate from a straight line connecting the start and the end of the movement; and (h) the lateral cursor position (*x*-coordinate) at the end of the reach movement were also considered.

An additional measure was extracted from the drop phase to indicate the completion quality of the prior intention. Discontinuities in the velocity profile toward the end of a movement indicate that an individual has corrected an impending error (Khan et al., 2006); therefore, the movement time following a discontinuity (or adjustment) can be used as an inverse measure of planning efficiency. To determine adjustments, we identified zero-order crossings of acceleration of the movement, which has been used in previous studies (Seidler et al., 2010; Rand and Stelmach, 2011). The time between the first secondary peak and movement offset was defined as adjustment time (ms). In all cases, these zero-order crossings always occurred after peak deceleration. Where no zero-order crossings were apparent, adjustment time was set to zero. We then calculated the period of adjustment time in percentage of the drop movement duration (Figure 3 – c).

#### 2.4.2 Statistical analysis

Each kinematic variable was entered into a separate 3 × 2 × 2 mixed ANCOVA, with the target side (left vs. right) and target fit (loose vs. medium vs. tight) being within-subject variables and age group (young vs. older) being the between-subject variable, and the participants’ self-report of daily mouse use being the covariate to control for the effect of mouse usage on the computer-based movement task.^1^ Where sphericity was violated, Greenhouse–Geisser correction was used. Simple effects analyses were applied when significant interactions were found. *Post hoc* analyses with Bonferroni-type correction were conducted when required, and the corrected significance level for each analysis was reported. Estimated marginal mean and standard error are reported for significant ANCOVA results. This procedure has been applied repeatedly throughout the analysis. The mean and standard deviation of each measure are reported in Table 1 for each group in each condition.

**Table 1:** Mean (*sd*) of all the kinematic variables in the reach and drop phase for each target side and fit of older and young participants.

Variables	Phase	Age group	Left			Right		
			Tight	Medium	Loose	Tight	Medium	Loose
Movement duration (ms)	Reach	Older	921 (224)	1,003 (192)	931 (202)	986 (213)	987 (189)	953 (189)
		Young	589 (68)	592 (82)	577 (66)	586 (78)	586 (70)	576 (90)
	Drop	Older	2,659 (1448)	1861 (988)	1851 (1583)	2,848 (1679)	1745 (776)	1,598 (719)
		Young	889 (247)	605 (105)	516 (77)	838 (220)	613 (86)	515 (75)
Peak velocity (pixel/ms)	Reach	Older	1743 (542)	1780 (608)	1783 (553)	1,666 (580)	1706 (551)	1,693 (553)
		Young	3,271 (655)	3,320 (666)	3,301 (643)	3,340 (678)	3,289 (568)	3,290 (642)
	Drop	Older	1,549 (564)	1,542 (575)	1,607 (695)	1,544 (616)	1,535 (507)	1,538 (487)
		Young	2,455 (528)	2,493 (522)	2,507 (477)	2,340 (507)	2,409 (525)	2,569 (562)
Peak acceleration (pixel/ms^2^)	Reach	Older	18,161 (8186)	18,311 (8913)	18,613 (8156)	17,095 (8856)	17,114 (8518)	17,903 (8768)
		Young	44,037 (11272)	44,616 (12024)	44,972 (11425)	44,265 (12058)	44,179 (10226)	44,256 (11223)
	Drop	Older	15,659 (8386)	15,799 (8561)	16,513 (10055)	16,212 (9128)	16,373 (7746)	16,231 (7593)
		Young	31,302 (8810)	31,843 (8711)	32,236 (8394)	28,650 (7950)	30,140 (8512)	32,323 (8672)
Time to peak acceleration (%)	Reach	Older	19.3 (15.1)	19.2 (12.8)	18.3 (12.3)	14.9 (9.8)	14.3 (9.6)	15.3 (9.3)
		Young	10.2 (2.4)	10.8 (3.1)	10.1 (2.0)	10.6 (2.5)	10.2 (2.2)	10.1 (1.9)
	Drop	Older	19.9 (12.5)	23.7 (14.1)	25.8 (13.5)	18.8 (10.4)	25.7 (15.0)	25.7 (12.1)
		Young	8.2 (3.7)	9.3 (2.8)	10.4 (2.9)	7.4 (3.0)	8.6 (2.2)	10.3 (3.1)
Deceleration period (%)	Reach	Older	68.2 (16.2)	71.9 (11.1)	73.4 (10.7)	75.7 (9.8)	74.3 (8.1)	74.9 (9.8)
		Young	80.2 (3.4)	80.0 (3.4)	80.1 (3.1)	79.6 (3.3)	80.4 (2.9)	80.3 (2.7)
	Drop	Older	76.8 (10.4)	71.8 (12.4)	68.8 (12.6)	77.6 (9.8)	67.1 (12.5)	66.5 (10.7)
		Young	84.2 (4.8)	79.6 (3.3)	76.6 (4.1)	82.9 (4.5)	80.7 (3.4)	77.6 (4.0)
Period of adjustment time (%)	Drop	Older	60.4 (14.3)	51.7 (15.7)	50.5 (12.9)	62.8 (12.0)	46.2 (11.7)	43.5 (16.3)
		Young	58.9 (8.3)	44.8 (7.9)	38.3 (8.4)	54.6 (9.2)	45.9 (7.9)	38.2 (8.5)

Similar to Wilmut and Wang (2019), adjustments in the reach movement deceleration period in accordance with prior intention (i.e., the final drop target side or size) are considered as evidence of planning for prior intentions. To examine the possible relationship between tailoring of a reach movement and prior intention for the target side, if a significant target side or target size effect was found, we calculated the difference in reach movement deceleration duration (%) as a proxy of prior intention planning (i.e., the degree to which an individual tailors a reach movement to the prior intention action). The relationships between reach movement adjustments and drop movement prior intention (adjustment time %) were examined using Pearson’s correlation and partial correlation to control for the chronological age. In addition to using averaged prior intention performance as an indicator of anticipatory planning ability, we also examined the relationship between the tailoring of the reach movement and the difference in prior intention performance between conditions. For multiple correlations, the alpha level was adjusted using Bonferroni correction.

If age-related changes were found in the prior intention, possible cognitive, sensorimotor and motor planning constraints were considered using hierarchical regressions – whether such age differences could be accounted for by choice reaction time (cognitive), pursuit rotor performance (sensorimotor), reach movement deceleration period (%; prior intention).

## 3 Results

First, the number of successful trials were entered into a 3 × 2 × 2 mixed ANOVA, with the target side (left vs. right) and target fit (loose vs. medium vs. tight) being within-subject variables and age group (young vs. older) being the between-subject variable. The main effect of target fit was significant [*F*(1.97, 169.31) = 26.65, *p* < 0.001, *η*^2^_p_ = 0.24], where tight fit (*M* = 7.81, *se* = 0.17) had a significantly smaller number of successful trials than medium (*M* = 8.74, *se* = 0.13, *p* < 0.001) and loose fit (*M* = 8.73, *se* = 0.17, *p* < 0.001). A significant age group difference was also found [*F*(1, 86) = 28.82, *p* < 0.001, *η*^2^_p_ = 0.25], where older adults (*M* = 7.72, *se* = 0.21) had a smaller number of successful trials than young adults (*M* = 9.14, *se* = 0.16). These results suggested that while the task seemed to be more challenging for older participants, tight fit was more difficult than medium and loose fit for both young and older participants. The main effect of target side [*F*(1, 86) = 0.01, *p* = 0.92] and interactions (all *F*s < 0.82, *p*s > 0.44) were not significant.

### 3.1 Movement kinematics

#### 3.1.1 Reach movement

Kinematics describing the overall movement – *movement duration and peak velocity*: In terms of *movement duration*, a significant main effect of age group was found [*F*(1, 85) = 238.70, *p* < 0.001, *η*^2^_p_ = 0.74], with older adults having a longer duration (*M* = 954 ms, *se* = 18 ms) than young adults (*M* = 590 ms, *se* = 14 ms). The target fit also had a significant main effect, *F*(1.87, 158.86) = 5.84, *p* = 0.004, *η*^2^_p_ = 0.06, where the reach duration for loose-fit (*M* = 758 ms, *se* = 12 ms) is significantly shorter than that for medium-fit (*M* = 790 ms, *se* = 13 ms; *p* < 0.001). There was no significant effect of the target side or any interactions (all *F*s < 3.0, *p*s > 0.05, *η*^2^_p_s < 0.04). Daily mouse use was a significant co-variate with participants used mouse more having shorter movement duration [*F*(1, 85) = 9.72, *p* = 0.002, *η*^2^_p_ = 0.10], but did not interact with other factors (all *F*s < 1.21, *p*s > 0.30, *η*^2^_p_s < 0.02).

In terms of *peak velocity*, a significant effect of age group was found, *F*(1, 85) = 164.87, *p* < 0.001, *η*^2^_p_ = 0.66, where older adults (*M* = 1748 px/s, *se* = 93 px/s) had a lower peak velocity than young adults (*M* = 3,290 px/s, *se* = 74 px/s). Other main effects and interactions are not significant (all *F*s < 0.76, *p*s > 0.38, *η*^2^_p_s < 0.01). The covariate daily mouse use did not have a significant effect or interact with other factors (all *F*s < 2.43, *p*s > 0.12, *η*^2^_p_s < 0.03).

Kinematics describing the ballistic phase – *peak acceleration and time to peak acceleration (%)*: In terms of the magnitude of reach movement *peak acceleration*, the main effect of the age group was significant [*F*(1, 85) = 153.43, *p* < 0.001, *η*^2^_p_ = 0.64], where young people had a higher peak acceleration (*M* = 44,167 px/s^2^, *se* = 1,283 px/s^2^) than older adults (*M* = 18,217 px/s^2^, *se* = 1,626 px/s^2^), but none of the other main effects or interactions was significant (all *F*s < 1.84, *p*s > 17, *η*^2^_p_s < 0.03). The covariate daily mouse use did not have a significant effect or interact with other factors (all *F*s < 1.84, *p*s > 0.17, *η*^2^_p_s < 0.03).

**Figure 4:**
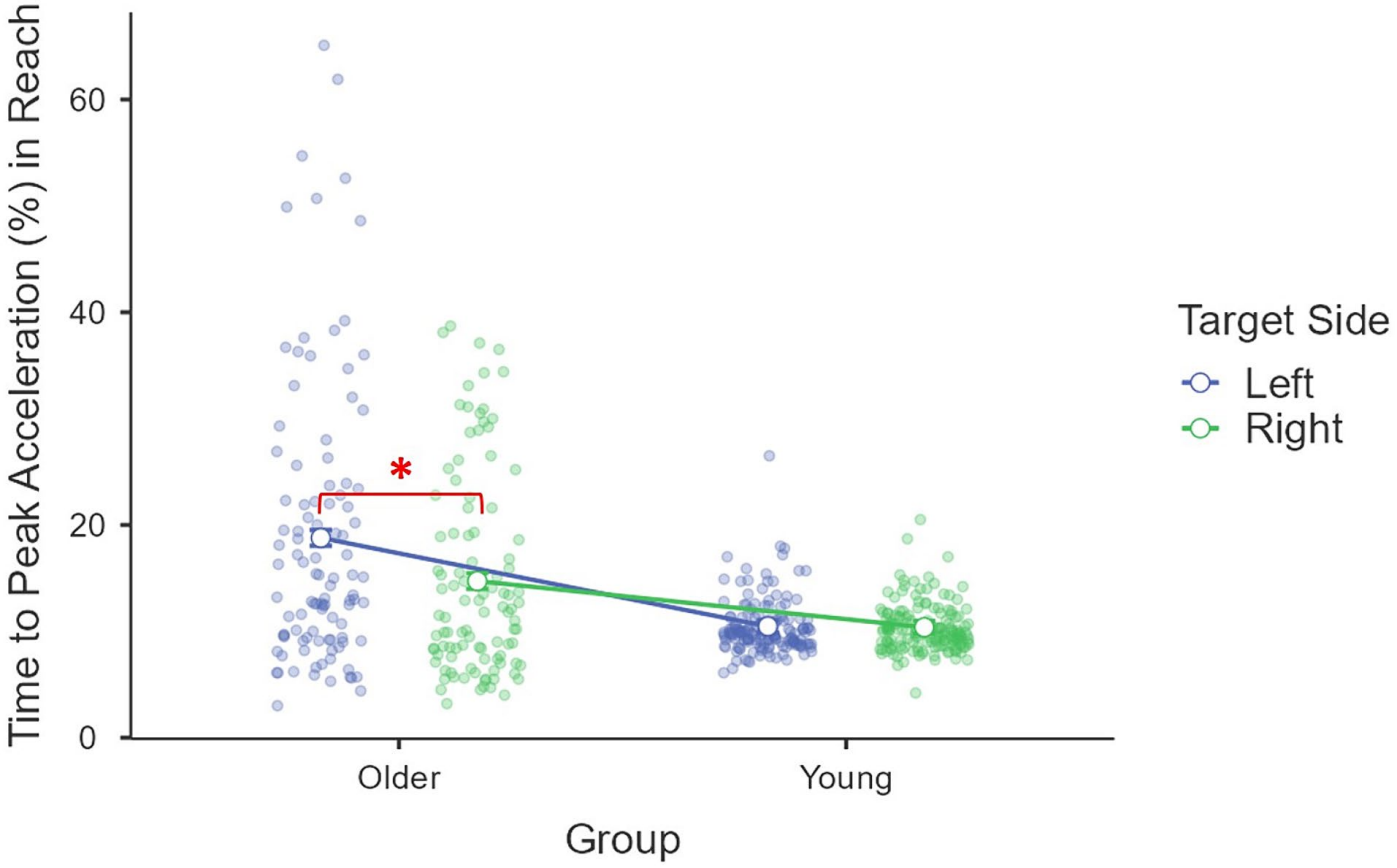
Interaction between age group and target side on reach movement time to peak acceleration (%).

In terms of *time to peak acceleration (%)*, a significant main effect of age group was found [*F*(1, 85) = 26.25, *p* < 0.001, *η*^2^_p_ = 0.24], where older adults (*M* = 16.8%, *se* = 1.0%) used a larger proportion of time to reach the peak acceleration than young adults (*M* = 10.4%, *se* = 0.8%). The main effect of the target side was also significant, *F*(1, 85) = 15.03, *p* < 0.001, *η*^2^_p_ = 0.15, that a larger proportion of time was used for targets on the left (*M* = 14.6%, *se* = 0.8%) than those on the right (*M* = 12.6%, *se* = 0.5%). This target side effect was moderated by age group [*F*(1, 85) = 11.65, *p* < 0.001, *η*^2^_p_ = 0.12; Figure 2] and driven by older adults [*F*(1, 85) = 21.52, *p* < 0.001, *η*^2^_p_ = 0.20] with a larger proportion of movement time being used to reach peak acceleration for targets on the left side (*M* = 18.7%, *se* = 1.2%) than those on the right side (*M* = 14.8%, *se* = 0.8%). The difference was not significant for young adults [*F*(1, 85) = 0.09, *p* = 0.76, *η*^2^_p_ < 0.01, left: *M* = 10.5%, *se* = 1.0%; right: *M* = 10.3%, *se* = 0.6%]. Other main effects and interactions were not significant (all *F*s < 0.53, *p*s > 0.56, *η*^2^_p_s < 0.01). The covariate daily mouse use did not have a significant effect or interact with other factors (all *F*s < 1.64, *p*s > 0.20, *η*^2^_p_s < 0.02) (Figure 4).

**Figure 5:**
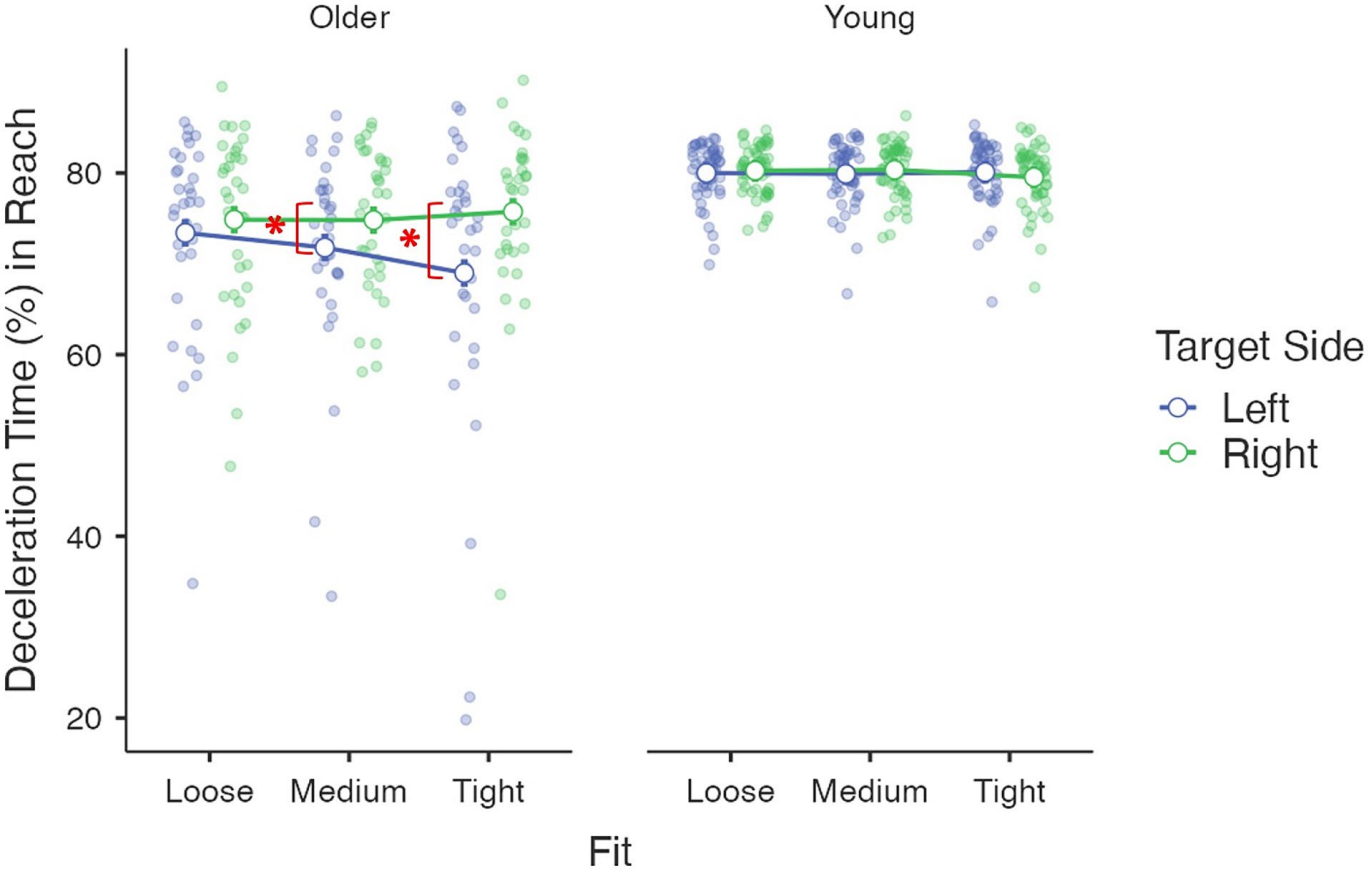
Interaction among age group, target side and fit on the reach movement deceleration period (%).

Kinematics describing the online phase – *deceleration time (%)*: The age group had a significant main effect, *F*(1, 85) = 32.04, *p* < 0.001, *η*^2^_p_ = 0.27. Young adults (*M* = 80.0%, *se* = 0.7%) spent a larger proportion of time decelerating than older adults (*M* = 73.2%, *se* = 0.9%). The main effect of the target side was also significant, *F*(1, 85) = 11.62, *p* = 0.001, *η*^2^_p_ = 0.12, where a longer deceleration period was observed for the targets on the right side (*M* = 77.6%, *se* = 0.5%) than those on the left side (*M* = 75.7%, *se* = 0.8%). Again, this target side effect was moderated by age group [*F*(1, 85) = 9.80, *p* = 0.002, *η*^2^_p_ = 0.10] and driven by older adults [*F*(1, 85) = 17.33, *p* < 0.001, *η*^2^_p_ = 0.17], with older people having a significantly longer deceleration period for the right side (*M* = 75.1%, *se* = 0.8%) than the left side targets (*M* = 71.4%, *se* = 1.2%). No significant difference was found for young adults [*F*(1, 85) = 0.03, *p* = 0.87, *η*^2^_p_ < 0.01, left: *M* = 80.0%, *se* = 1.0%; right: *M* = 80.1%, *se* = 0.6%]. A three-way interaction among the target side, fit and age group was also significant [*F*(1.61, 136.40) = 4.53, *p* = 0.02, *η*^2^_p_ = 0.05; Figure 3]. The target-side by fit interaction was only significant for older adults [*F*(2, 85) = 3.72, *p* = 0.03, *η*^2^_p_ = 0.08], where an elongated deceleration period for right-side targets than for left-side targets was found for tight (*p* < 0.001) and medium fits (*p* = 0.03) but not loose fit (*p* = 0.40). The simple effect of fit was only significant for targets on the left side [*F*(2, 85) = 5.03, *p* = 0.009, *η*^2^_p_ = 0.11] with a shorter deceleration period for a tight fit than that for a loose fit (*p* = 0.008). The covariate daily mouse use did not have a significant effect or interact with other factors (all *F*s < 0.93, *p*s > 0.33, *η*^2^_p_s < 0.02) (Figure 5).

Kinematics describing the movement path – *path length*, *maximum path deviation on the x-coordinate and the lateral cursor position at the end of the movement:* In terms of *path length*, a significant age group difference was found [*F*(1, 85) = 46.45, *p* < 0.001, *η*^2^_p_ = 0.35], where young participants (*M* = 448.26 px, *se* = 3.92 px) a longer path than older participants (*M* = 444.70 px, *se* = 4.96 px). Other effects were not significant, all *F*s < 3.90, *p*s > 0.05.

For *the maximum path deviation on the x-coordinate*, target side had a significant main effect [*F*(1, 85) = 28.88, *p* < 0.001, *η*^2^_p_ = 0.25], and this effect was driven by older participants [*F*(1, 85) = 13.53, *p* < 0.001, *η*^2^_p_ = 0.14], where the deviation was toward the right for left side targets (*M* = 22.22 px, *se* = 3.82 px) and toward the left for right side targets (*M* = −7.20 px, *se* = 3.28 px; *p* < 0.001). There was no significant difference for young participants (*p* = 0.20). Other effects were not significant (all *F*s < 2.65, *p*s > 0.07).

For *the lateral cursor position at the end of the reach movement*, the target side effect was also significant [*F*(1, 85) = 24.84, *p* < 0.001, *η*^2^_p_ = 0.23], and this effect was driven by older participants [*F*(1, 85) = 25.73, *p* < 0.001, *η*^2^_p_ = 0.23]. Older participants ended their reach movement more to the right if targets were on the left (*M* = 1.50 px, *se* = 0.51 px) and more to the left for targets on the right (*M* = −2.44 px, *se* = 0.48 px). There was no significant difference for young participants (*p* = 0.87). Other effects were not significant (all *F*s < 1.87, *p*s > 0.15).

#### 3.1.2 Drop movement

Kinematics describing the overall movement – *movement duration and peak velocity*: In terms of *movement duration*, the main effect of age group was significant, *F*(1, 85) = 95.01, *p* < 0.001, *η*^2^_p_ = 0.53. Older adults had a longer duration (*M* = 2054 ms, *se* = 109 ms) than young adults (*M* = 688 ms, *se* = 86 ms). The main effect of target fit was also significant, *F*(1.45, 123.19) = 84.86, *p* < 0.001, *η*^2^_p_ = 0.50, where the tight fit (*M* = 1798 ms, *se* = 94 ms) was longer than medium (*M* = 1,200 ms, *se* = 56 ms) and loose fit (*M* = 1,114 ms, *se* = 73 ms; both *p*s < 0.001). The difference between medium and loose fits was not significant (*p* = 0.08). The interaction between fit and age group was also significant, *F*(1.45, 123.19) = 20.61, *p* < 0.001, *η*^2^_p_ = 0.20. The age group difference was significant for all levels of fit, with older adults showing longer movement durations than young adults (all *F*s > 60.02, *p*s < 0.001, *η*^2^_p_s > 0.41). For both older and young adults, tight fit had a longer duration than medium and loose fit (all *p*s < 0.002), however, with a much larger effect size for older [*F*(2, 85) = 50.92, *p* < 0.001, *η*^2^_p_ = 0.55] than for young adults [*F*(2, 85) = 8.54, *p* < 0.001, *η*^2^_p_ = 0.17]. The main effect of the target side and interactions were not significant (all *F*s < 2.57, *p*s > 0.09, *η*^2^_p_s < 0.03). Daily mouse use was a significant co-variate with participants used mouse more having shorter movement duration [*F*(1, 85) = 4.71, *p* = 0.033, *η*^2^_p_ = 0.05]. It did not interact with other factors (*F*s < 1.57, *p*s > 0.21, *η*^2^_p_s < 0.02).

In terms of *peak velocity*, a significant main effect of age group was found, *F*(1, 85) = 85.47, *p* < 0.001, *η*^2^_p_ = 0.50, where young adults (*M* = 2,466 px/s, *se* = 61 px/s) had a greater peak velocity than older adults (*M* = 1,546 px/s, *se* = 77 px/s). A significant effect of fit was also found, *F*(1.86, 158.30) = 3.30, *p* = 0.043, *η*^2^_p_ = 0.04. Pairwise comparisons revealed a greater peak velocity for a loose fit (*M* = 2054 px/s, *se* = 52 px/s) than a tight fit (*M* = 1971 px/s, *se* = 53 px/s; *p* = 0.024), and all other differences were not significant (both *p*s > 0.17). The main effect of target side and interactions were not significant either (all *F*s < 2.28, *p*s > 0.10, *η*^2^_p_s < 0.03). The covariate daily mouse use did not have a significant effect or interact with other factors (all *F*s < 2.64, *p*s > 0.10, *η*^2^_p_s < 0.04).

Kinematics describing the ballistic phase – *peak acceleration and time to peak acceleration (%)*: In terms of the magnitude of drop movement *peak acceleration*, the main effect of the age group was significant [*F*(1, 85) = 90.75, *p* < 0.001, *η*^2^_p_ = 0.52], where young people had a higher peak acceleration (*M* = 31,173 px/s^2^, *se* = 976 px/s^2^) than older adults (*M* = 15,989 px/s^2^, *se* = 1,237 px/s^2^). A significant main effect of fit was also found [*F*(1.72, 146.52) = 4.21, *p* = 0.021, *η*^2^_p_ = 0.05], where tight fit (*M* = 22,934 px/s^2^, *se* = 831 px/s^2^) had lower peak acceleration than loose fit (*M* = 24,305 px/s^2^, *se* = 826 px/s^2^, *p* = 0.003). Other effects were not significant, and the covariant and interactions were not significant either (all *F*s < 2.32, *p*s > 0.10, *η*^2^_p_s < 0.03).

In terms of *time to peak acceleration (%)*, the main effect of age group was significant, *F*(1, 85) = 133.75, *p* < 0.001, *η*^2^_p_ = 0.61, where older adults (*M* = 22.9%, *se* = 0.9%) used more time to reach peak acceleration than young adults (*M* = 9.3%, *se* = 0.7%). The effect of fit was also significant, *F*(1.95, 166.00) = 14.62, *p* < 0.001, *η*^2^_p_ = 0.15, where a smaller proportion of movement time was used to reach peak acceleration for a tight fit (*M* = 13.5%, *se* = 0.6%) than that for medium (*M* = 16.7%, *se* = 0.9%) and loose fit (*M* = 18.0%, *se* = 0.7%; both *p*s < 0.002). The difference between medium and loose fit was not significant (*p* = 0.46). Other main effects and interactions were not significant (all *F*s < 3.07, *p*s > 0.05, *η*^2^_p_s < 0.04). Daily mouse use was a significant covariate where participants used mouse more frequently had a smaller proportion of movement time used to accelerate [*F*(1, 85) = 5.04, *p* = 0.027, *η*^2^_p_ = 0.06], but the interactions with other factors were not significant (all *F*s < 1.43, *p*s > 0.24, *η*^2^_p_s < 0.02).

**Figure 6:**
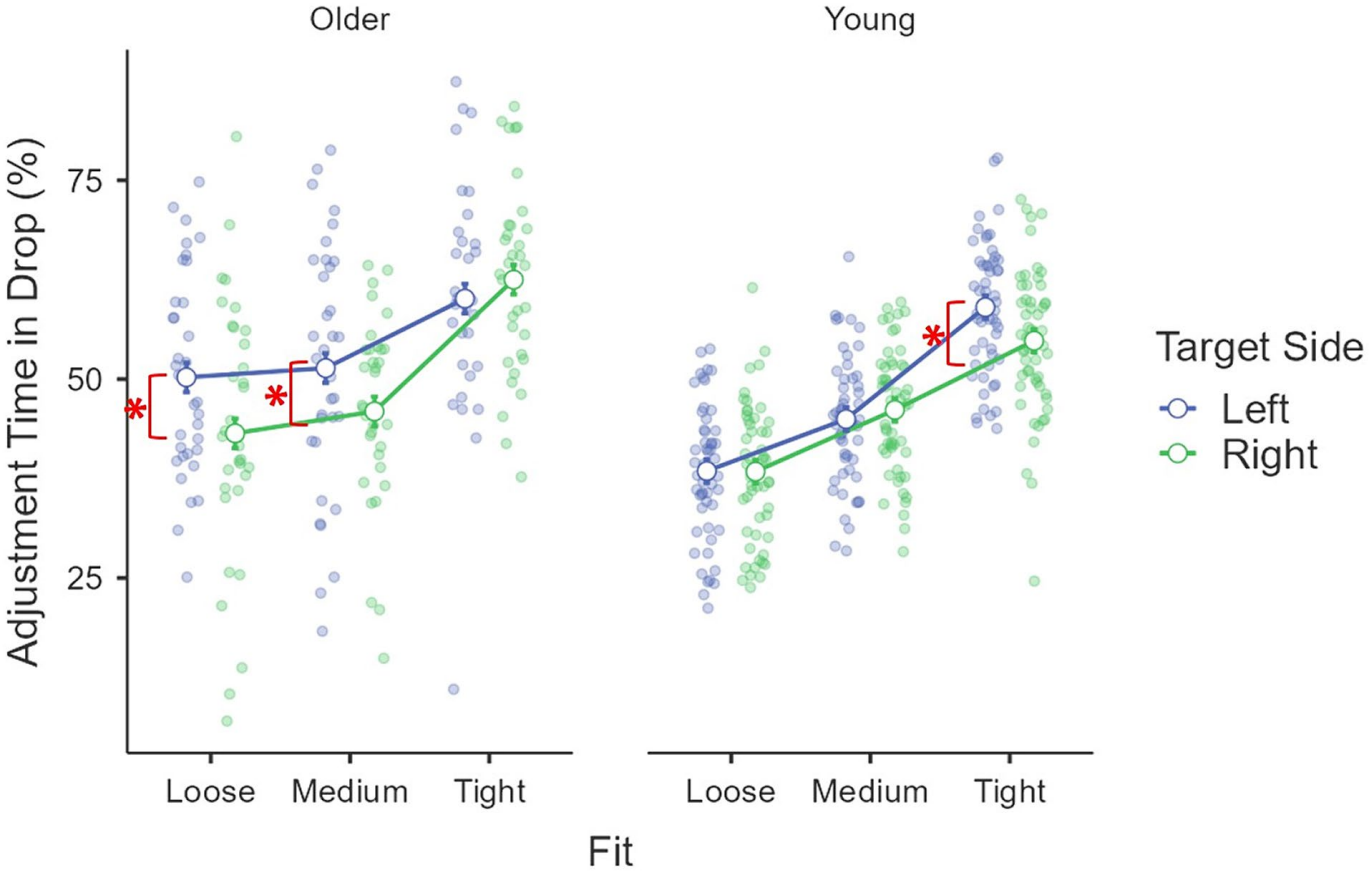
Interaction among age group, target side and fit on the drop adjustment time (%).

Kinematics describing the online phase – *deceleration time (%)*: the main effect of age group was significant, *F*(1, 85) = 68.13, *p* < 0.001, *η*^2^_p_ = 0.45, where older adults (*M* = 71.6%, *se* = 0.8%) used less time to decelerate than young adults (*M* = 80.2%, *se* = 0.6%). The main effect of target fit was significant, *F*(1.94, 164.85) = 49.24, *p* < 0.001, *η*^2^_p_ = 0.37, where a larger proportion of time was used to decelerate for a tight fit (*M* = 80.4%, *se* = 0.6%) than a medium (*M* = 74.8%, *se* = 0.8%) and a loose fit (*M* = 72.4%, *se* = 0.7%; both *p*s < 0.001). The difference between medium and loose fit was also significant (*p* = 0.006). The effect of fit was moderated by age group, *F*(1.94, 164.85) = 3.10, *p* = 0.049, *η*^2^_p_ = 0.04. Pairwise comparisons revealed that the difference between medium (*M* = 80.0%, *se* = 1.0%) and loose (*M* = 77.0%, *se* = 0.8%) was only significant for young adults (*p* = 0.006) but not older adults (*p* = 0.38). A three-way interaction was found for fit × age group × target side, *F*(1.98, 168.36) = 5.05, *p* = 0.008, *η*^2^_p_ = 0.06 (Figure 6), where the difference between left (*M* = 71.9%, *se* = 1.4%) and right side (*M* = 67.4%, *se* = 1.4%) was only significant for older adults’ medium fit [*F*(1, 85) = 11.71, *p* < 0.001, *η*^2^_p_ = 0.12]. The covariant of daily mouse use and its interactions with other factors were not significant (all *F*s < 2.27, *p*s > 0.10, *η*^2^_p_s < 0.03).

Kinematics describing the movement path – *path length*: Target fit had a significant main effect, *F*(1.73, 147.10) = 9.98, *p* < 0.001, *η*^2^_p_ = 0.11, where tight fit (*M* = 500.99 px, *se* = 6.49 px) had a longer path length than medium (*M* = 479.91 px, *se* = 5.04 px; *p* = 0.004) and loose fit (*M* = 479.49 px, *se* = 6.31 px; *p* < 0.001). A significant age group difference was also found [*F*(1, 85) = 87.48, *p* < 0.001, *η*^2^_p_ = 0.51], where older participants (*M* = 535.29 px, *se* = 8.05 px) a longer path than young participants (*M* = 438.31 px, *se* = 6.35 px). Other effects were not significant, all *F*s < 3.90, *p*s > 0.05.

Kinematics describing the prior intentions – *adjustment time (%)*: The main effect of age group was significant, *F*(1, 85) = 15.17, *p* < 0.001, *η*^2^_p_ = 0.15, where older adults (*M* = 52.2%, *se* = 1.0%) used more time to adjust than young adults (*M* = 47.0%, *se* = 0.8%). The main effect of fit was also significant, *F*(1.77, 150.30) = 127.73, *p* < 0.001, *η*^2^_p_ = 0.60, where longer adjustment was found for a tight fit (*M* = 59.2%, *se* = 0.9%) than a medium (*M* = 47.1%, *se* = 0.9%) than a loose fit (*M* = 42.5%, *se* = 1.0%; all *p*s < 0.001). The effect of the target side was also significant, *F*(1, 85) = 4.23, *p* = 0.042, *η*^2^_p_ = 0.05, where the left side drop (*M* = 50.7%, *se* = 0.9%) had a longer adjustment time than the right side drop (*M* = 48.5%, *se* = 0.8%). A three-way interaction was also found for age group × fit × target side, *F*(1.94, 164.98) = 10.23, *p* < 0.001, *η*^2^_p_ = 0.11 (Figure 6). When considering the effect of fit, while the simple main effect was significant across all conditions (all *F*s > 8.93, *p*s < 0.001, *η*^2^_p_s > 0.17), the difference between medium and loose was not significant for older adults on either left or right side (both *p*s > 0.33), but significant between other conditions for older adults (all *p*s < 0.003) and among all conditions for young adults (all *p*s < 0.001). When considering the effect of the target side, significant differences were found for older adults’ medium [*F*(1, 85) = 5.20, *p* = 0.025, *η*^2^_p_ = 0.06] and loose fits [*F*(1, 85) = 10.73, *p* = 0.002, *η*^2^_p_ = 0.11] and young adults’ tight fit [*F*(1, 85) = 5.46, *p* = 0.022, *η*^2^_p_ = 0.06], where a longer adjustment time was observed for the left side targets than the right side targets in each comparison. The covariant of daily mouse use and its interactions with other factors were not significant (all *F*s < 2.93, *p*s > 0.09, *η*^2^_p_s < 0.04).

### 3.2 Planning for prior intention

#### 3.2.1 Relationship between tailoring of a reach movement and prior intention for the target side

**Figure 7:**
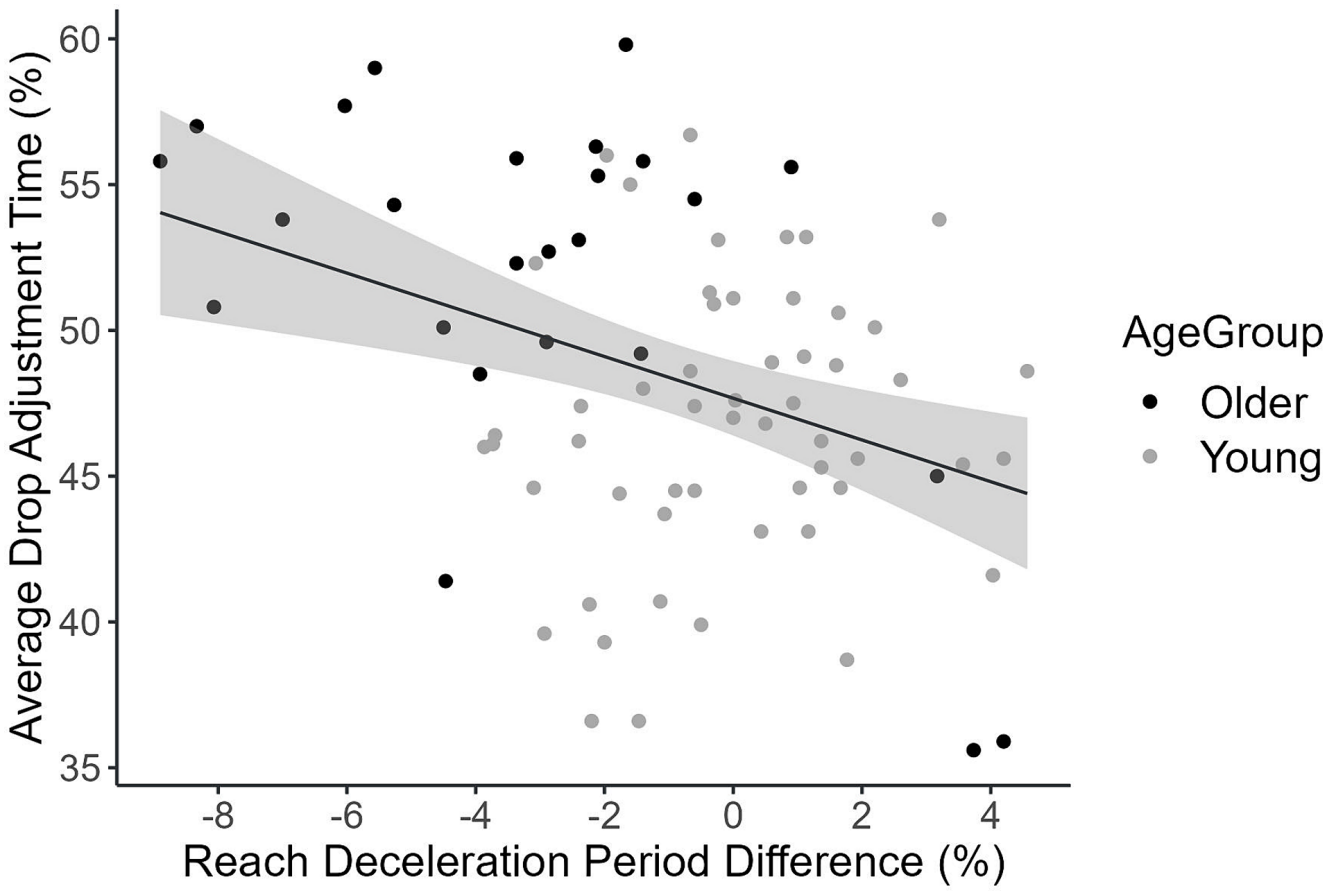
Association between deceleration period difference between left and right side (%) and the average drop adjustment time (%) plotted for older and young participants.

In the reach movement, the time to peak acceleration (%), the deceleration time (%), maximum path deviation on the *x*-coordinate, and lateral cursor position at the end of the reach movement were tailored as a function of the target side. Thus, we calculated the difference in the time to peak acceleration (%) (ballistic phase), the deceleration duration (%) (online phase) and the two path feature measures between left and right as adjustment indices measuring the degree to which an individual tailors a reach movement to the prior intentions. Nine participants were excluded from further analyses due to outlying indices of differences. Pearson’s correlation analyses revealed that the adjustments in the reach movement time to peak acceleration [*r*(77) = 0.33, *p* = 0.003] and deceleration period [*r*(77) = −0.31, *p* = 0.006] were significantly associated with the prior intention performance (i.e., drop movement adjustment time %; Figure 7), but not maximum path deviation on the *x*-coordinate [*r*(77) = 0.11, *p* = 0.33] or the lateral cursor end position [*r*(77) = 0.08, *p* = 0.48]. However, after controlling for the chronological age, only the reach movement deceleration period difference was still significantly correlated with drop movement adjustment time [*r*(77) = −0.24, *p* = 0.035]. A positive correlation was found between the difference in deceleration time (%) between left and right side targets and the difference in prior intention performance between left and right [*r*(77) = 0.29, *p* = 0.009, after controlling for the chronological age]. Thus, only the deceleration period was included in further analyses.

#### 3.2.2 Possible age-related constraints: cognitive function, sensorimotor function, and motor planning

We further explored whether age-related differences in prior intentions (adjustment time %) can be explained by participants’ choice reaction time, pursuit rotor performance, and their reach movement deceleration time adjustments. Hierarchical regression models show that, for prior intention (adjustment time %), participants’ choice reaction time and pursuit rotor performance are both significant predictors. On top of these, the reach movement deceleration difference was a significant predictor and accounted for additional variance in the drop movement prior intentions. Together, these variables accounted for the age group difference in prior intention (Table 2).

**Table 2:** F-change, *R*^2^-change, beta, *t*-value and associated *p*-values from the hierarchical regression when predicting adjustment time (%).

	*F* change	*R^2^* change	*p* – model comparison	Variables	*β*	*t*	*p*
Model 1	8.23	0.10	0.005	Choice reaction time	<0.001	2.87	0.005
Model 2	5.10	0.06	0.03	Choice reaction time	<0.001	0.79	0.43
				+ Pursuit rotor	−0.07	−2.26	0.03
Model 3	4.76	0.05	0.03	Choice reaction time	<0.001	0.33	0.74
				+ Pursuit rotor	−0.06	−2.01	0.05
				+ Reach phase: deceleration period difference	−0.49	−2.18	0.03
Model 4	0.12	0.001	0.74	Choice reaction time	<0.001	0.25	0.80
				+ Pursuit rotor	−0.05	−1.01	0.32
				+ Reach phase: deceleration period difference	−0.46	−1.95	0.05
				+ Age group	0.01	0.34	0.74

## 4 Discussion

The current study investigated anticipatory planning for multiple prior intentions and its age-related changes using a computer-based reach-to-click task. During the task, participants completed two-step movements, reaching toward a disk from the home button and then dropping the disk with different prior intentions. Adjustments in the initial reach movement depending on the final drop action parameters are an indication of planning for prior intentions. This is the first time multiple prior intentions are considered in one sequential movement, namely the side of the target and the size of the target (i.e., fit). First, we conducted kinematic analyses of the computer mouse cursor trajectories during the reach-to-click task and examined the adjustments in the initial reach movement and the following drop movement as a function of the onward action parameters for both young (18–22) and older adults (40–68), where the adjustments in the reach movement is considered planning for prior intentions. Second, we considered whether the age-related changes in the onward action performance could be accounted for by individuals’ cognitive, sensorimotor and anticipatory planning for prior intentions.

### 4.1 Kinematic analysis of reach movement – planning for prior intention (side)

Similar to studies that used the real-world reach-to-grasp paradigm, we included five kinematic measures of the initial reach movement: the movement duration and peak velocity describing the features of the entire reach movement, peak acceleration and the time to peak acceleration describing the early ballistic phase under feedforward control, and deceleration period for the online phase under feedback control. Except for the peak velocity and peak acceleration, all the measures showed adjustments based on the prior intention of movement direction (target side) or fit difficulty (target size).

Movement duration was the only measure differentiated for the fit difficulty in the reach movement, where both young and older adults used less time reaching toward the disk when the target size was large, and the fit was presumably easier (reach duration: medium > loose). This is in line with real-world movement that shorter reach movement was found for those followed by easier onward actions (Egmose and Køppe, 2018: lift > fit > throw; Wilmut and Wang, 2019: tight fit > loose fit). Reach movement peak velocity did not change according to the prior intentions of movement direction or fit precision/difficulty. This is also similar to real-world movements (Weir et al., 1998; Johnson-Frey et al., 2004; Gigliotti et al., 2020), where significant differences were often driven by involving a throw and/or a lift, which are considered very easy or difficult, in the onward actions (Wilmut et al., 2013; Wilmut and Wang, 2019). While the reach movement paths differed on the x-coordinate according to the onward phase target side, suggesting that participants differed in the paths during the reach movement to set the state for the onward drop action. However, such differentiation did not contribute to the onward intention performance. When considering accumulated movement path, young participants traveled longer path length than older participants in the reach phase, and older participants traveled longer in the drop phase. Regarding target fit, tight fit had a longer path length in the drop phase than medium and loose, which suggests that the accumulated path length is associated with drop movement difficulty. The extra path length traveled by older participants in the drop phase gave insight into the intensity of the compensation in travel distance elicited by task difficulty. However, similar to movement duration, this measure is not sensitive enough to reflect differentiation and adjustments for onward intentions.

When considering the ballistic phase, older adults used more time to reach peak acceleration for a left-side than a right-side drop, though the magnitudes of peak acceleration were similar for both sides. Wilmut and Wang (2019) found that only young people (20s) adjusted their reach movement and used more time to reach peak acceleration for lift than for fit (both tight and loose). Lift is often considered the most challenging onward action in prior intention studies. Although, so far, no study has considered movement direction as prior intentions, left-forward movement is indeed more challenging than right-forward movement, which is shown in the literature (Dillen et al., 2005; Hertzum and Hornbæk, 2010; Stewart et al., 2013) and our data. In our data, the final adjustment time for the left-forward drop was longer than that for the right-forward drop, indicating that a larger velocity discontinuity, and thus more correction, was required to complete the final drop movement toward the left side compared to the right side. Differences in the time to peak acceleration may show very early adjustments in the ballistic phase of reach movements for onward actions. Interestingly, this was only observed among older adults. The early stage movement kinematics are believed to represent one’s proactive strategies as a part of the feedforward process (Flanagan and Wing, 1997), thus such adjustments at a very early stage of movement suggested that older adults perceived the differences in movement direction and included such consideration into their internal model of movement, though not necessarily accurate. The age-related changes will be discussed in more detail below.

When considering the online control phase, older people spent more time decelerating for the right than the left-side targets, in particular when they were tight and medium fits. However, young people did not show such adjustments. This is contrary to our hypothesis, where left-forward movement was assumed to be more challenging than right-forward movement. Previous studies compared other intentions found a longer deceleration period in reach movement for more challenging prior intentions (lift) than easier ones (throw; Marteniuk et al., 1987; Weir et al., 1998; Johnson-Frey et al., 2004, Exp. 1 and 3). In some studies, when only the levels of fit difficulty were considered, young people spent more time decelerating during the reach movement for a tight fit than for a loose fit (Wilmut et al., 2013; Wilmut and Wang, 2019), though in other studies, there was no difference in reach phase deceleration for different fits/places among healthy young adults (Weir et al., 1998; Johnson-Frey et al., 2004; Gigliotti et al., 2020). Our data showed that older adults “planned” more for an easier onward action. The deceleration period in reach phase was considered as evidence for planning/adjusting for different onward intentions, as more challenging movements taking longer to plan (decelerate; Thompson et al., 2007). However, in our study, when the drop action is easier, e.g., toward right-forward direction, the deceleration period was longer, which seems counterproductive. Interestingly, a similar pattern was observed among young children. When studying the development of prior intention planning among children, the youngest group (4–5-year-olds) showed a similar profile to our older adults that a smaller proportion of time was used to decelerate for a tight fit (55.8%) than a loose fit (57.3%, though simple main effect analysis did not reach significance level; Wilmut et al., 2013). In the same study, a similar deceleration period between tight and loose fit was found for 6–9 years of age children. Only for the oldest group (10–11+), a longer deceleration period for tight than loose fit emerged. This seems to suggest that, when considering planning for final fit size, the computer-based task for older adults may be similar to the real-world grasp task for young children of 4–5 years of age. For young adults, the computer-based task is similar to 6–9 years of age completing the real-world task. Thus, the counterproductive adjustments among older adults were probably due to task difficulty. When individuals face particularly challenging tasks, such as real-world grasp for young children aged 4–5 years or computer-cursor task for older adults, even onward intentions were considered and early movement adjusted accordingly, such adjustments may not necessarily facilitate the completion of onward actions. Indeed, in this study, the counterproductive adjustment among older adults was only observed in the challenging tighter fits (tight and medium) rather than the easier loose fit.

An important question to ask here was whether such counterproductive adjustment could be considered “planning” that would lead to more efficient onward actions. The analysis showed a negative correlation between adjustment in the reach movement (deceleration period difference between left and right) and quality of onward fit movement (the adjustment time). This negative relationship between the two measures is in line with literature suggesting that tailoring reach movement (longer deceleration period) leads to more efficient final movement (Wilmut et al., 2013; Wilmut and Barnett, 2014; Wilmut and Wang, 2019). However, it should be noted that in the current study, the reach movement tailoring measure can be negative, which means a smaller value does not only mean participants did not differentiate the prior intentions but can also mean that the planning might be counterproductive. Indeed, the more they decelerated for right-forward, which is considered less challenging, than for left-forward fit, the longer adjustment time was observed in the onward action. In addition to using averaged prior intention performance as an indicator of the anticipatory planning ability, we also examined the relationship between the difference in deceleration time (%) between left and right side targets and the difference in prior intention performance between left and right, and there was a significant positive correlation after controlling for the chronological age. Ideally, more adjustment would be expected for more challenging actions, leading to better onward actions and resulting in performance similar to easier actions (a negative association). Thus, the positive association identified in the current data provided further evidence that the adjustment may be counterproductive. Despite the fact that older adults made counterproductive adjustments, our data showed that the measure of changes in the deceleration period was an effective index of anticipatory planning for prior intentions of movement direction. This is the first time movement direction was examined in an anticipatory planning task and found to be a valid prior intention. However, it should be noted that the prior intention of target fit was only evident in the overall movement duration but not in the deceleration period or other kinematic measures, which suggests that participants were not planning for the different levels of fit during the reach movement. More adjustments for target fit were observed in the drop movement kinematics.

Compared to young individuals, older participants demonstrate modifications in their motor planning, which can be attributed to feedforward-feedback mechanisms. In our dataset, these modifications were evident in both the early stage (time to peak acceleration %) and later stage (deceleration time %) of the reach movement. Early stage movement kinematics reflect proactive strategies within the feedforward process, while later stage movement kinematics represent reactive strategies as part of the feedback process (Poirier et al., 2020). Our data revealed that during onward intention planning, early stage adjustments (time to peak acceleration %, left > right) indicated that older participants perceived different demands between left- and right-side drop movements, and their internal feedforward model was accurate. However, the later stage adjustment (deceleration time %, left < right) was counterproductive, suggesting that online feedback-driven correction was inaccurate and implemented unwanted impediment to the movement. This is in line with studies that found that feedback processes become unreliable with healthy aging, while the feedforward process remains unaffected (Boisgontier and Nougier, 2013; Helsen et al., 2016; Wolpe et al., 2016; Vandevoorde and Orban De Xivry, 2019; Poirier et al., 2020). However, unlike previous research (Poirier et al., 2020), our data did not demonstrate strong enough compensation from the feedforward process for the less effective feedback control, as the final onward performance remained suboptimal for left-side targets compared to right-side targets. This may be attributed to the difficulty of the two-step anticipatory motor planning task in our study (one-step movement tasks were used in studies mentioned above), where older participants exhibited counterproductive feedback adjustments with an opposite effect (longer deceleration for an easier condition).

### 4.2 Kinematic analysis of the drop movement (fit)

We also conducted analyses of the five kinematic measures for the drop movement (onward action), and all five measures showed adjustment as a function of the final fit (tight vs. medium vs. loose), which shows motor intentions for the intrinsic property of the target size. For both young and older adults, tight fit had longer movement duration, lower peak velocity, and shorter time to reach a lower peak acceleration. The only measure that showed age group difference was the deceleration period that young adults differentiated among all the fits (tight > medium > loose), whereas older adults only differentiated tight from medium and loose. This is in line with previous studies where the onward action kinematics were examined (Weir et al., 1998; Johnson-Frey et al., 2004; Wilmut and Barnett, 2014; Gigliotti et al., 2020). It should be noted that all previous studies used real-world 3D movement tasks, showing that the adjustment for immediate motor intentions is highly consistent across real-world and computerized tasks. In our task, the deceleration phase adjustment also showed an age-group difference, suggesting the current computer task may be more sensitive in terms of detecting age-related alterations, even considering the onward action as a single-step movement.

### 4.3 Multiple intentions in one movement sequence

The primary goal of this study is to investigate adjustments for multiple intentions in one movement sequence. According to Jeannerod (2006), the physical attributes of the object (e.g., size and texture) are considered an intrinsic property, and the relational attributes with respect to oneself (e.g., direction and distance) are considered extrinsic property. Our study considered both object intrinsic property (target size) and extrinsic property (target side, movement direction) and found that they are primarily considered or adjusted for at different phases of the reach-to-click movement. Across the analyses of kinematic measures, where adjustments were found for reach movement, it is primarily based on the extrinsic property of movement direction (left-forward vs. right-forward), whereas drop movement adjustment was based on the intrinsic property of target size (tight vs. medium vs. loose). Although movement direction was not examined in previous studies, literature considered different levels of difficulty of the same movement (tight vs. loose fit) often found that the effect of fit difficulty was more likely to be observed during the drop/fit movement (motor intention) than during the reach movement (prior intention; Johnson-Frey et al., 2004; Gigliotti et al., 2020). Our data showed that kinematic adjustments for extrinsic properties, such as movement direction, appear at an earlier stage of sequential movement than that for intrinsic properties, such as target size. Compared to intrinsic properties, extrinsic properties determine human-object interactions, where the adjustments are for a larger scale (direction and distance) than adjustments for target size. This result suggests a step-by-step planning/control strategy, and the temporal dynamics of the anticipatory planning “resolution” is from configural/larger scale (e.g., movement direction) to fine-detailed/smaller scale fit size (e.g., grip aperture).

More interestingly, such effects were more conspicuous among older participants, though the adjustments during reach movement were not necessarily effective for the planning of prior intentions, they showed more adjustments than young people. This seems to suggest that planning for multiple prior intentions is more challenging as we grow older, and older people can perceive such task constraints as less affordable given their mouse control capacity, hence adjusting for the movement direction and fit difficulty step-by-step, which may be a way to compensate for adjusting multiple requirements in one go. However, knowing the need for planning or adjustment is different from planning effectively. As aforementioned, their adjustment for movement direction was counterproductive. Young people did not adjust their reach movement for prior intentions but still completed the onward actions in a more efficient manner than older people. This may be attributable to the computer-based task and the use of a computer mouse, which can be incredibly challenging for older people but simple for young people, who do not even need to adjust in advance to be able to complete the task smoothly.

### 4.4 Age constraints: cognitive, sensorimotor, planning for prior intention

An age-related decline in the final movement quality (increased adjustment time of drop movement) was observed. We considered whether our measured constraints of general cognitive capacity, sensorimotor function and anticipatory planning for prior intentions would influence the onward action performance. One’s general cognitive capacity, which was measured using choice reaction time, and sensorimotor function, which was measured using the Pursuit Rotor task with a focus on eye-hand coordination, are both predictive of one’s action performance. This is in line with studies demonstrating associations between cognitive abilities and anticipatory motor planning (Stöckel et al., 2017; Wang et al., 2020).

The difference in this study is that we used choice reaction time as a single measure for one’s cognitive function rather than a number of tasks measuring multiple components of human cognition. Choice reaction time is believed to account for the core ability in heterogeneous cognitive tasks (Deary and Der, 2005). According to the processing speed hypothesis of cognitive aging, reaction time elongates with age, mainly due to the slowing down of information processing, and accounts for a substantial proportion of the age-related variance (or decline) in higher cognitive functions such as memory, reasoning, and executive functions. Also, we used choice reaction time to the “Go” trials from a Stop Signal Task rather than the response time of the current movement planning task. This is because we sought to use the choice reaction time as an index of general cognitive function that is sensitive to aging, whereas reaction time (or initiation time) in movement planning/control tasks is believed to involve both the selection of motor goals and the planning or preparation for the movement (Hertzum and Hornbæk, 2010; Wong et al., 2015; Delmas et al., 2018), which may confound the results.

In terms of the sensorimotor function, we considered one’s eye-hand coordination using a computerized pursuit rotor task. Eye-hand coordination represents a sophisticated perceptual-motor skill that involves the integration of various sensorimotor components and declines in advanced aging (Guan and Wade, 2000; Boisseau et al., 2002). Pursuit rotor task has been used to successfully identify age-related declines in eye-hand coordination, with older participants exhibiting lower time-on-target values compared to young participants (Durkina et al., 1995). We used a computerized pursuit rotor task in the current study, which examines one’s eye-hand coordination by moving a computer mouse to control the cursor on the screen to track a moving target. The sensorimotor functions required in this task are very similar to our computerized motor planning task. Indeed, we found a lower time-on-target value for older adults than young adults, which is in line with previous studies where a decline in eye-hand coordination was found in the healthy aging (Durkina et al., 1995; Boisseau et al., 2002; de Picker et al., 2014; Marini et al., 2019). Moreover, this eye-hand coordination performance is a significant predictor of prior intention proficiency in addition to the cognitive function.

In the regression model, we entered the index of planning for prior intention (reach movement deceleration period difference) in the last step. On top of the cognitive and sensorimotor functions, one’s ability to plan for prior intentions was a significant predictor that accounted for additional variances in the final movement quality. This means that one’s ability to plan for prior intention plays a unique role in motor control that could not be simply explained by one’s general cognitive or sensorimotor function. After considering all these three factors, the age group difference in the onward action quality no longer existed, which shows that the age-related changes in movement quality are indeed constrained by one’s cognitive, sensorimotor and anticipatory planning abilities. These results also demonstrated the validity and sensitivity of the current computerized task and the kinematic measures based on mouse cursor tracking, which is a portable and low-cost paradigm that can be widely used as a screening tool for motor aging out of the lab. So far, most of the studies of movement kinematics have been conducted in a lab setting relying on motion capture systems, such as Vicon or Qualisys. Despite the cost of equipment, motion tracking systems often require a controlled environment for the set-up, calibration and even lightning control for optical motion capture. In this case, the computer-based task and mouse tracking techniques are more affordable, portable, and accessible to a wider population, e.g., senior subjects and/or those with difficulties in mobility to visit the lab.

### 4.5 Limitations

There are several limitations in the current study. First, when considering planning for prior intentions, we use pre-assumed task or action difficulty to examine whether individuals adjusted their initial movements accordingly. However, we never considered individuals’ perceived task difficulty or movement affordability. While not necessarily accurate, such perceptions or predictions one made would affect one’s movement adjustment. Considering one’s subjective task difficulty gives us access to further investigate the mechanisms underlying movement adjustment – whether counterproductive adjustments are rooted in erroneous evaluation of the action affordability. Second, the current computerized task seems to be a successful pilot, but there are improvements to make. One is that the current task seemed to be very challenging for older adults, which may be one of the reasons they made counterproductive adjustments. While research compared multiple cursor control devices (Touch Sensitive Screen, Mouse, Graphics Tablet, Touchpad, Trackball, and Joystick) found that the computer mouse is the easiest to use (Dillen et al., 2005; Lee and Bang, 2013; MacKenzie, 2018) and the most user-friendly, even for children and elders (Hertzum and Hornbæk, 2010), there is additional visual feedback processing of the mouse cursor in the computer-based movement task, which may be more demanding for older than young people. Thus, in future studies, we will modify the task parameters to be more adaptive for older people. Third, in the current study, we did not have a set of real-world motor planning data from the same group of participants using a comparative setup. Last, the sample size and age range were not ideal. The data was collected during Covid-19 when social distance and travel restrictions were applied. Inviting older participants to the lab on a university campus was very challenging. Future studies may consider a wider age range with a larger and more distributed sample.

### 4.6 Conclusion

In summary, this study investigated planning for multiple prior intentions in a computer-based reach-to-click task among young and older adults and found that the extrinsic property of onward action (movement direction) is considered at an earlier stage of sequential movement than the intrinsic property (target size fit difficulty), in particular for older people. Moreover, we found that age-related changes in motor performance on onward actions were constrained by general cognitive and sensorimotor coordination decline, and on top of that, how effective one’s anticipatory planning for prior intention is accounted for extra effect that could not be explained by general cognitive and sensorimotor functions. Age-related decline in anticipatory motor planning is manifested in counterproductive movement profiles that may lead to suboptimal motor performance in accomplishing prior intentions.

## Data availability statement

The original contributions presented in the study are included in the article/Supplementary material, further inquiries can be directed to the corresponding author.

## Ethics statement

The studies involving humans were approved by Duke Kunshan University Institutional Review Board. The studies were conducted in accordance with the local legislation and institutional requirements. The participants provided their written informed consent to participate in this study.

## Author contributions

SZ: Data curation, Formal analysis, Project administration, Visualization, Writing – original draft. KW: Conceptualization, Methodology, Writing – review & editing. KZ: Conceptualization, Writing – review & editing. SW: Conceptualization, Formal analysis, Funding acquisition, Methodology, Project administration, Resources, Supervision, Visualization, Writing – review & editing.

## Footnotes

^1^We also selected young and older participants with a daily mouse use rating of 1 (rarely use a computer mouse) and ran mixed-measures 3 × 2 × 2 ANOVAs on each kinematic variable. Similar results of the significance tests were found.
